# Whole-genome resequencing using next-generation and Nanopore sequencing for molecular characterization of T-DNA integration in transgenic poplar 741

**DOI:** 10.1186/s12864-021-07625-y

**Published:** 2021-05-06

**Authors:** Xinghao Chen, Yan Dong, Yali Huang, Jianmin Fan, Minsheng Yang, Jun Zhang

**Affiliations:** 1 Forest Department, Forestry College, Hebei Agricultural University, Baoding, China; 2 Hebei Key Laboratory for Tree Genetic Resources and Forest Protection, 071000 Baoding, PR China

**Keywords:** Transgenic poplar 741, T-DNA, Integration site, Copy number

## Abstract

**Background:**

The molecular characterization information of T-DNA integration is not only required by public risk assessors and regulators, but is also closely related to the expression of exogenous and endogenous genes. At present, with the development of sequencing technology, whole-genome resequencing has become an attractive approach to identify unknown genetically modified events and characterise T-DNA integration events.

**Results:**

In this study, we performed genome resequencing of Pb29, a transgenic high-resistance poplar 741 line that has been commercialized, using next-generation and Nanopore sequencing. The results revealed that there are two T-DNA insertion sites, located at 9,283,905–9,283,937 bp on chromosome 3 (Chr03) and 10,868,777–10,868,803 bp on Chr10. The accuracy of the T-DNA insertion locations and directions was verified using polymerase chain reaction amplification. Through sequence alignment, different degrees of base deletions were detected on the T-DNA left and right border sequences, and in the flanking sequences of the insertion sites. An unknown fragment was inserted between the Chr03 insertion site and the right flanking sequence, but the Pb29 genome did not undergo chromosomal rearrangement. It is worth noting that we did not detect the *API* gene in the Pb29 genome, indicating that Pb29 is a transgenic line containing only the *BtCry1AC* gene. On Chr03, the insertion of T-DNA disrupted a gene encoding TAF12 protein, but the transcriptional abundance of this gene did not change significantly in the leaves of Pb29. Additionally, except for the gene located closest to the T-DNA integration site, the expression levels of four other neighboring genes did not change significantly in the leaves of Pb29.

**Conclusions:**

This study provides molecular characterization information of T-DNA integration in transgenic poplar 741 line Pb29, which contribute to safety supervision and further extensive commercial planting of transgenic poplar 741.

**Supplementary Information:**

The online version contains supplementary material available at 10.1186/s12864-021-07625-y.

## Background

Poplar is one of the most widely distributed tree species owing to its rapid growth and strong adaptability to environmental changes [1–3]. It is one of the important industrial timber species that is widely used in the paper-making industry and panel processing. However, with the continuous increase of poplar planting area, the ensuing insect attack has become more and more serious, which has brought huge losses to forestry production [4]. In order to reduce the economic losses caused by insect pests, decrease the need for chemical pesticides, and protect the ecological environment, the cultivation of insect-resistant transgenic varieties is particularly important [5]. Transgenic technology is used commercially for growing trees in China, which was the first country to commercialize transgenic poplar.

At the same time, the possible impact of transgenic technology on humans and ecology is still unclear. Therefore, China, like most other countries and regions in the world, is still very cautious about the application and supervision of transgenic technology, requiring that the research and experiment, environmental release and commercial production of genetically modified organisms (GMOs) all require safety certificates provided by relevant departments [6]. Inheritance and expression stability of exogenous genes is a prerequisite for commercial application of transgenic plants, which depends on the molecular characteristics of T-DNA integration into the host genome [7]. Because of the randomness and non-replicability of T-DNA integration, the molecular information of T-DNA integration becomes the specific marker of transgenic plants, which is conducive to the identification and supervision of different transgenic lines. The genome sequence (genetic material) of a transgenic plant has been altered due to the insertion of T-DNA through genetic engineering [8]. Several studies have shown that the molecular characterization of T-DNA integration, including T-DNA sequence, insertion position, copy number and flanking sequences of the insertion site, will affect the expression of transgenes. In hybrid poplar, the transgene inactivation is always the result of transgene repetition [9]. Fladung et al. analyzed three unstable 35S-rolC transgenic aspen lines, and the results showed that transgene expression may be highly variable and unpredictable when the transgenes are present in the form of repeats [10]. In GFP-transgenic barley, when the insert is proximate to the highly repetitive nucleolus organizer region (NOR) on chromosome 7, the expression of the transgene is completely silent, while fluorescent expression appears in other regions [11]. Kumar et al. indicated that the host genome can control the expression of a foreign gene, and AT-rich regions may play a role in defense against foreign DNA [9]. Furthermore, T-DNA insertion often leads to expected and unexpected changes at transcriptional, protein and metabolic levels in transgenic plants, which potentially affects food/feed quality and safety [12, 13]. Therefore, clarifying the molecular characterization data of T-DNA integration such as T-DNA copy number and insertion site locations is particularly important for risk assessors and regulators of transgenic plants.

There are many methods for locating the insertion sites of foreign genes in transgenic plants, most of which are based on polymerase chain reaction (PCR) amplification; these include thermal asymmetric interlaced PCR [14], inverse PCR [15], and adapter-ligated PCR [16]. Although these methods have been successfully applied to transgenic plants of species such as *Arabidopsis thaliana* [17] and rape [18], they are prone to false-positives, and are also time-consuming, laborious, and poorly reproducible. In recent years, with the continuous development of sequencing technology, next-generation sequencing (NGS) has been widely used for genome sequencing because of its high throughput capability, low cost, and accurate results. NGS has been successfully used to locate T-DNA insertion sites in transgenic soybean [19], rice [20], and birch [21]. However, the NGS reads are too short to accurately locate all of the T-DNA insertion sites in transgenic plants with complex T-DNA integration patterns or genomes. By contrast, third-generation sequencing technology, developed by Oxford Nanopore Technologies and PacBio, can produce longer reads, which can overcome the limitations of NGS such as short reads and bias due to GC content, although the accuracy is relatively low. Therefore, by combining NGS with third-generation sequencing technology, we can accurately and efficiently analyze overall genomic changes due to T-DNA mutations.

Poplar 741 is an excellent cultivar of the section Leuce Duby that was cultivated after two hybridizations in 1974. The hybridized combination is [*P. alba* L. × (*P. davidiana* Dode. + *P. simonii* Carr.)] × *P. tomentosa* Carr [22]. Transgenic poplar 741, which was cultivated by Hebei Agricultural University and the Institute of Microbiology of the Chinese Academy of Sciences, was obtained by *Agrobacterium*-mediated transformation of the expression vector containing *BtCry1AC* gene and arrowhead proteinase inhibitor (*API*) gene into poplar 741 [23]. According to national standards for transgenic animals and plants, transgenic poplar 741 has been certified safe after environmental impact and production tests and were planted commercially from 2002 to 2007. Pb29 is a high-resistance line of transgenic poplar 741. It carries two insect-resistant genes (*BtCry1AC* and *API*) in theory and shows high levels of resistance to lepidopteran pests, such as *Hyphantria cunea* and *Clostera anachoreta* [4, 23]. However, no molecular analysis of T-DNA integration in transgenic poplar 741 has been performed. In this study, we performed whole-genome resequencing of transgenic poplar 741 using NGS and Nanopore sequencing, and analyzed the copy number and insertion sites of the T-DNA as well as the flanking sequences at the T-DNA integration site. Our results obtained the molecular characterization data of T-DNA integration in transgenic poplar 741 line Pb29, which can provide precise information for safety supervision and contribute to further extensive commercial planting of transgenic poplar 741.

## Results

### Results of NGS analysis

**Fig. 1:**
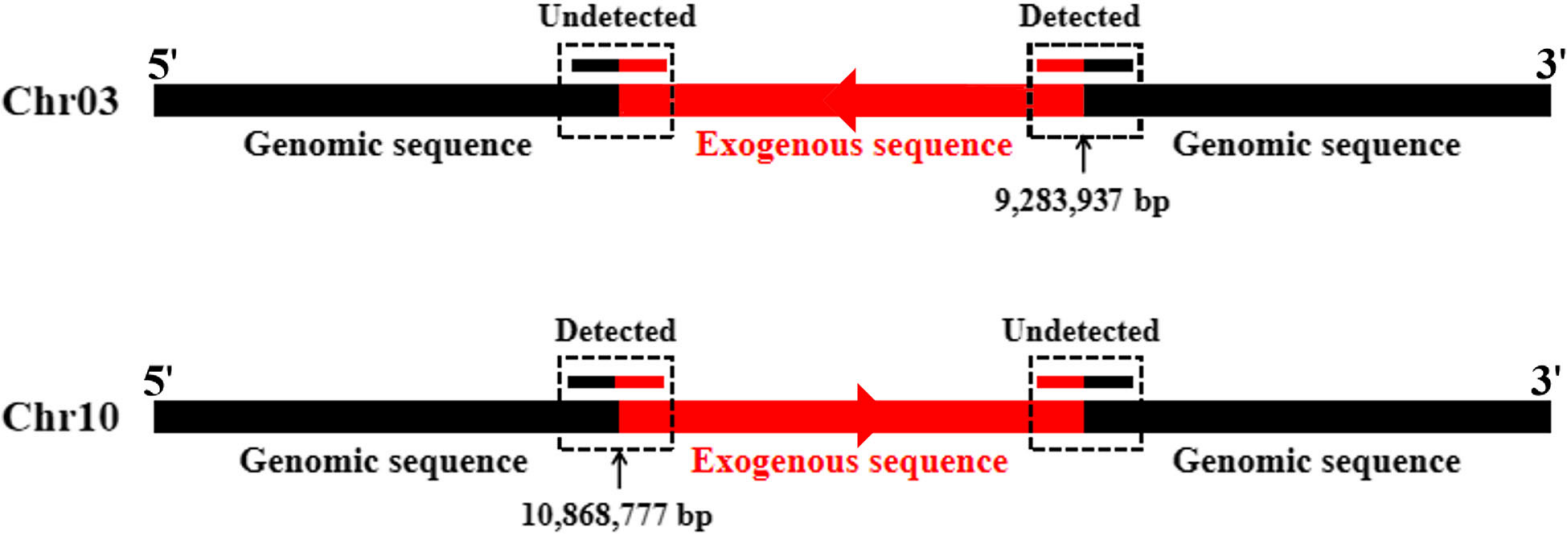
The detection results of T-DNA insertion sites obtained using NGS. Detected / Undetected indicates that the junction reads (reads containing both T-DNA and flanking genomic sequences) in the box with black dotted line were identified or not identified in NGS results

After performing quality-control checks, a total of 52.3 million clean reads for transgenic poplar 741 line Pb29 were obtained from the raw reads, corresponding to more than 30× coverage of the *Populus trichocarpa* reference genome (https://www.ncbi.nlm.nih.gov/genome/98). More than 92% of the sequencing data had Phred-like quality scores ≥30, indicating that the data were high quality (Table S1). After sequence alignment, nine junction reads on chromosome 03 (Chr03), and four on Chr10, were identified in the Pb29 genome sequence, indicating that there are two T-DNA insertion sites in the Pb29 genome (Table S2). Based on the physical positions of the junction reads, one insertion site is located at 9,283,937 bp on Chr03, and the other at 10,868,777 bp on Chr10. T-DNA is inserted in the reverse direction on Chr03, and in the forward direction on Chr10. However, further analysis revealed that only unilateral junction reads could be detected at both T-DNA insertion sites; ideally, junction reads should be detected on both sides of each insertion site (Fig. 1).

### Confirmation of insertion sites and directions using PCR amplification

**Fig. 2:**
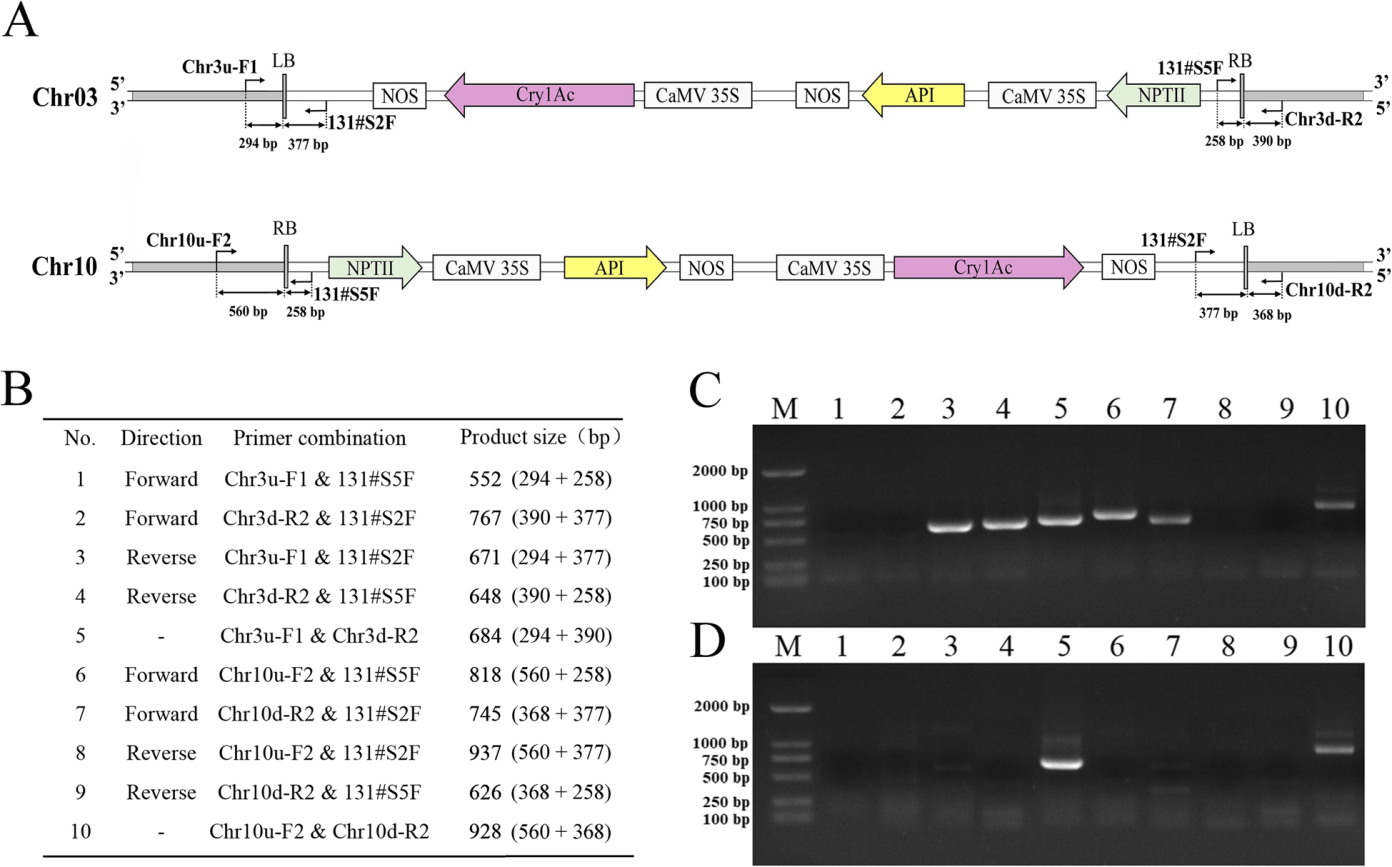
PCR verification of the insertion sites and directions of the T-DNA obtained by NGS in Pb29. **a** Schematic diagram of PCR primer design for verifying the insertion sites and directions of the T-DNA. LB: left border; RB: right border. **b** The primer combinations and product size for verifying the insertion sites and directions. Each number represents a primer combination. **c** The results of PCR amplification of genomic DNA of Pb29. **d** The results of PCR amplification of genomic DNA of poplar 741

To verify the accuracy of the T-DNA insertion sites and directions, we designed 6 primers based on the flanking sequences of the T-DNA insertion sites and the T-DNA sequence (Fig. 2a), and amplified the genomic DNA of poplar 741 and Pb29 using different primer combinations (Fig. 2b). The results of PCR amplification revealed that the PCR runs using primer combinations 3, 4, 6, and 7 generated products with a single band for Pb29 in Fig. 2c, whereas no products were amplified for poplar 741 in Fig. 2d. When primer combinations 1, 2, 8, and 9 were used in the PCR, amplified bands were not produced for Pb29 or poplar 741, indicating that T-DNA was indeed inserted into Chr03 in the reverse direction and into Chr10 in the forward direction, thus verifying the NGS results. Meanwhile, the target band was observed after PCR runs using primer combinations 5 and 10 for both Pb29 and poplar 741, indicating that Pb29 is a heterozygous mutant created via T-DNA insertion (Fig. 2c; Fig. 2d).

### Results of Nanopore sequencing analysis

To further verify the NGS results and determine whether chromosomal rearrangement occurred in the Pb29 genome due to T-DNA insertion, we used the third-generation sequencing technology developed by Oxford Nanopore Technologies to resequence the whole genomes of poplar 741 and Pb29. More than 96% of the clean reads of both poplar 741 and Pb29 mapped to the *P. trichocarpa* reference genome, corresponding to 40× and 39× coverage of the reference genome, respectively. The depth of coverage was evenly distributed across both poplar 741 and Pb29 chromosomes, indicating that the genomic DNA of poplar 741 and Pb29 was sequenced in a random manner (Fig. S1).

**Fig. 3:**
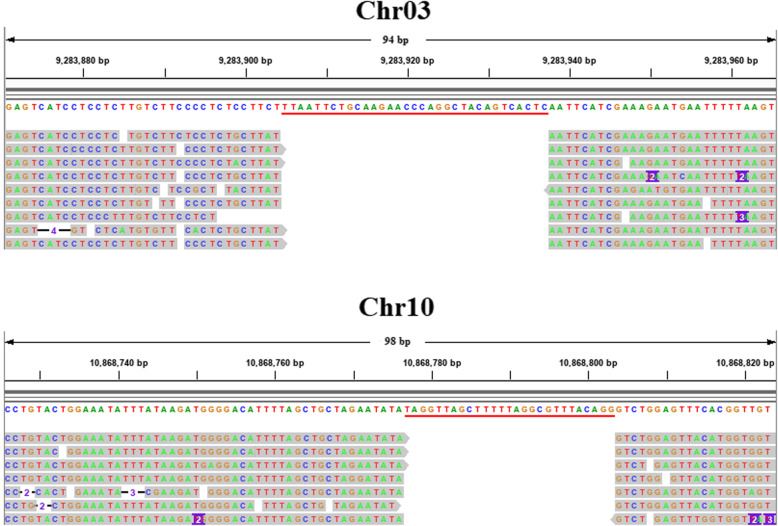
Visual analysis of junction reads obtained by Nanopore sequencing using IGV software. The discontinuous sequences are part of the reads obtained by Nanopore sequencing, and the continuous sequence is derived from the *P. trichocarpa* reference genome, with information on its length and chromosome location at the top. The base sequences marked with the red line are the gaps that are not aligned to the *P. trichocarpa* reference genome

The BAM file generated by comparing all junction reads with the *P. trichocarpa* reference genome was imported into Integrative Genomics Viewer (IGV) software for visual analysis. All junction reads only mapped to Chr03 or Chr10, and there was a gap between reads on both chromosomes. The two gaps, each formed by a T-DNA insertion that disrupted part of the genome sequence, matched the two T-DNA insertion sites in the Pb29 genome exactly. The two T-DNA insertion sites in the Pb29 genome are located at 9,283,905–9,283,937 bp on Chr03 and 10,868,777–10,868,803 bp on Chr10, consistent with the detection results obtained using NGS (Fig. 3).

Compared with the *P. trichocarpa* reference genome, evidence of many Structural variation (SV) events was seen in the genomes of both poplar 741 and Pb29, most of which were deletions or insertions of chromosome segments (Fig.S2). After removing the regions representing SV events of the same type at the same positions in the poplar 741 and Pb29 genomes, SV events > 1 kb are regarded as chromosomal rearrangements in the Pb29 genome caused by T-DNA insertion. However, we did not detect this type of event, indicating that the insertion of T-DNA did not cause large chromosomal rearrangements in the Pb29 genome.

### T-DNA and flanking sequence analysis

**Fig. 4:**
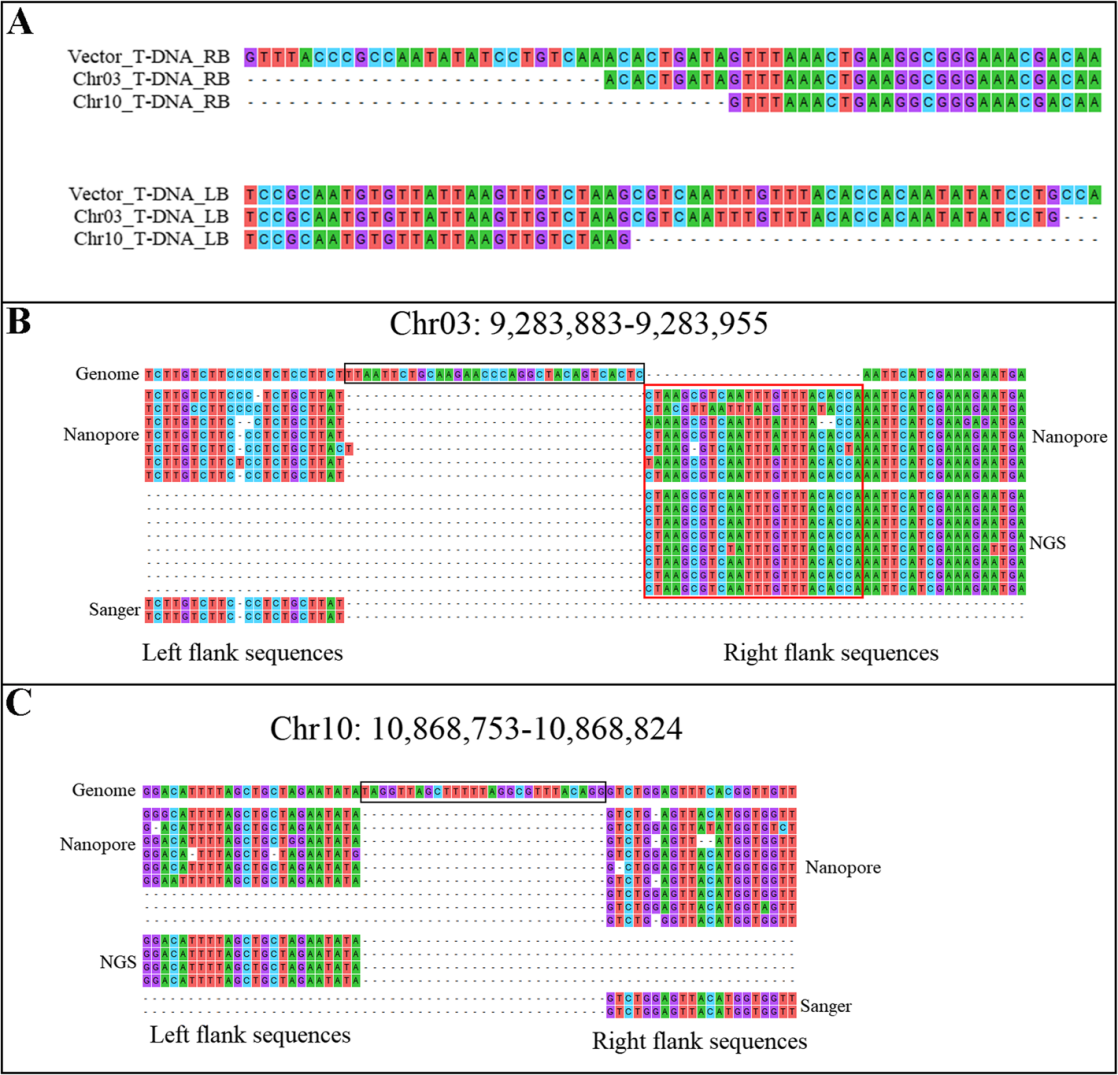
Analysis of the left and right border sequences of T-DNA and the flanking sequences of the insertion sites in the Pb29 genome. **a** Analysis of the left and right T-DNA border sequences in both insertion sites. Vector_T-DNA: T-DNA on the vector; Chr03_T-DNA: T-DNA inserted on chromosome 03; Chr10_T-DNA: T-DNA inserted on chromosome 10; RB: T-DNA right border; LB: T-DNA left border. **b** Analysis of flanking sequences of the both T-DNA insertion sites. The box with black outline is the base deletions occurred in the Pb29 genome sequence and the box with red outline is the base insertions occurred in the Pb29 genome sequence

**Fig. 5:**
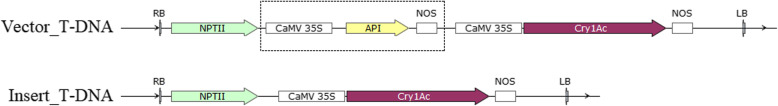
Analysis of inserted T-DNA sequences and vector T-DNA sequence. The black dashed box is the missing 35S-API-Nos expression component; LB: left border; RB: right border

Because Nanopore sequencing can be used to obtain longer reads, some junction reads contained complete T-DNA sequences. The complete T-DNA sequences at the two insertion sites were extracted and compared with the vector sequence. The results showed that the left and right border sequences of the T-DNA inserted on Chr03 were missing 26 and 3 bp, respectively, whereas the left and right border sequences of the T-DNA inserted on Chr10 were missing 35 and 34 bp, respectively (Fig. 4a). It is worth noting that the 35S-API-Nos expression component was not detected in the T-DNA sequences at either insertion site; furthermore, both T-DNA sequences are exactly the same, indicating that the expression component of the *API* gene was not lost during the transformation process. Rather, it was not present in the expression vector in *Agrobacterium* before transformation (Fig. 5).

We compared isolated flanking sequences with the *P. trichocarpa* reference genome and found that fragments had been deleted from the flanking sequences at both insertion sites, as T-DNA insertion damaged the genome sequence at those sites (box with black outline in Fig. 4b and Fig. 4c). The genome sequence at the T-DNA insertion sites on Chr03 and Chr10 was missing 33 and 27 bp, respectively, consistent with the results of the alignment analysis (Fig. 3). A short fragment (24 bp in length) was found between the T-DNA insertion site and the right flanking sequence on Chr03 in the Pb29 genome; this fragment could not be mapped to the *P. trichocarpa* reference genome (box with black outline in Fig. 4b). We analyzed the clean reads from poplar 741 found that reads mapped to the same positions essentially had the same sequences as the corresponding sections of the *P. trichocarpa* genome (Fig. S3), indicating that the 24-bp fragment did not arise from the difference between genomes but was instead caused by the insertion of an unknown fragment during the T-DNA integration process.

### Analysis of the expression levels of genes located near the insertion sites

**Fig. 6:**
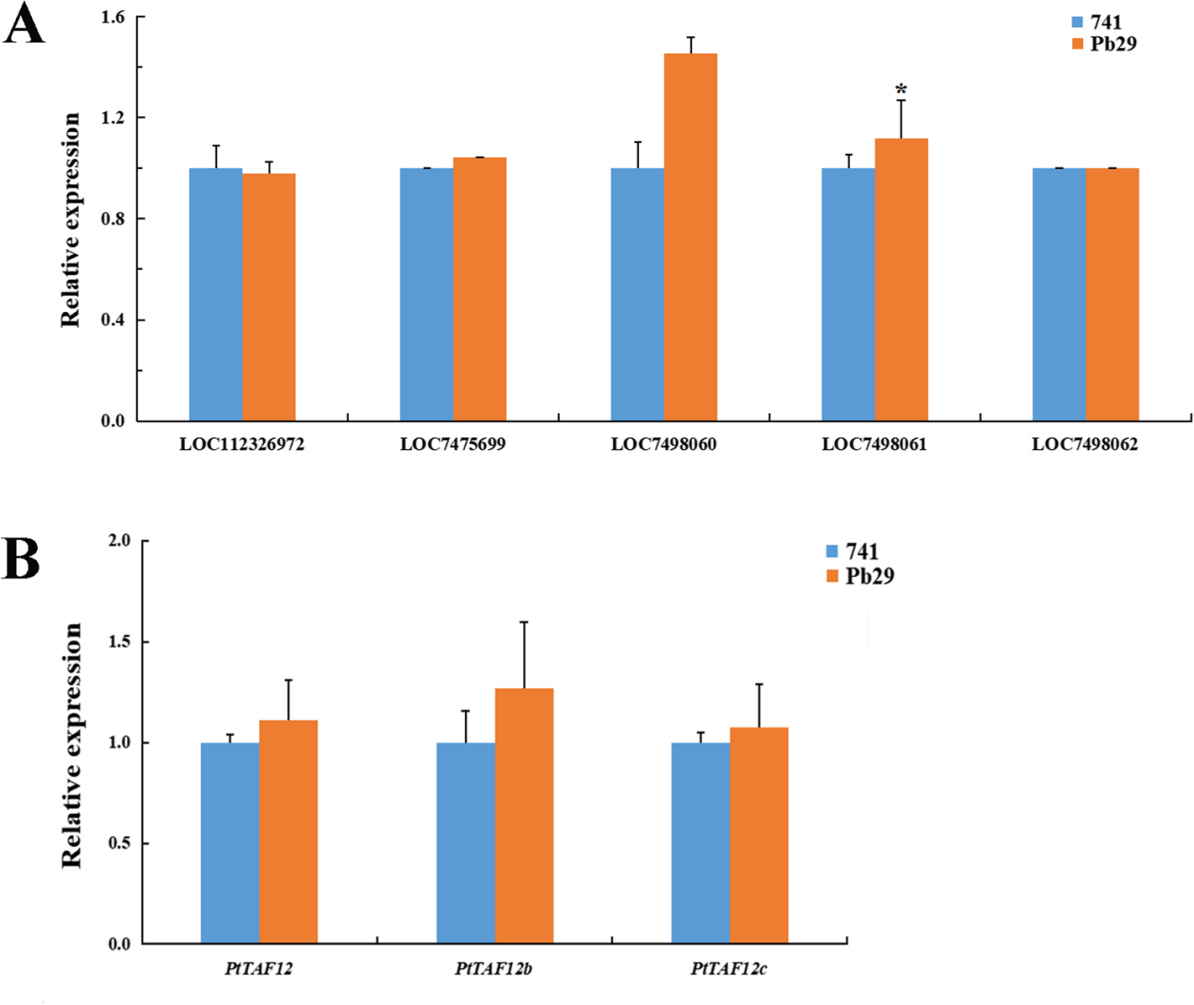
Relative expression analysis of genes in healthy and mature leaves of mature tree of poplar 741 and Pb29 using RNA-seq. **a** Analysis of the relative expression levels of genes located near the insertion sites. **b** The relative expression of the genes encoding TAF12 protein. The FPKM values of genes in poplar 741 and Pb29 obtained by RNA-seq were changed by the same fold to analyze the expression changes of the genes in Pb29 relative to those in poplar 741. All data are presented as the mean ± SEM. (*, *P* < 0.05)

The genes within 20 kb upstream and downstream of the two T-DNA insertion sites were detected based on the genome annotation file of *P. trichocarpa*. The results showed that T-DNA was inserted 9466 bp downstream of the LOC112326972 gene and 8137 bp upstream of the LOC7475699 gene on Chr03, and 15,621 bp downstream of the LOC7498060 gene and 1543 and 11,914 bp upstream of the LOC7498061 and LOC7498062 genes, respectively, on Chr10 (Table 1). Fragments Per Kilobase Million (FPKM) values associated with the transcriptome data were used to compare the expression levels of the five neighboring genes. The results showed that except for the LOC7498061 gene, the expression levels of the other four genes in Pb29 leaves did not change significantly, indicating that the insertion of T-DNA did not significantly affect the expression levels of these four genes. The LOC7498061 gene is located closest to the T-DNA insertion site; its expression level was significantly upregulated in Pb29 leaves, indicating that the insertion of T-DNA in Pb29 affects gene expression within a certain range (Fig. 6a).

**Table 1:** The genes located near the insertion sites

Insertion location	Neighboring gene(< 20 kb)		Genomic location
Chr03:9283905–9,283,937	Upstream	LOC112326972	Chr03:9261716:9274439
	Downstream	LOC7475699	Chr03:9292074:9294391
Chr10:10868777–10,868,803	Upstream	LOC7498060	Chr10:10848741:10853156
	Downstream	LOC7498061	Chr10:10870346:10873516
		LOC7498062	Chr10:10880717:10883716

### Analysis of the *TAFs* gene family

According to the results of whole-genome resequencing analysis, the T-DNA insertion site on Chr03 (9,283,895–9,283,937 bp) is located within the first exon of the LOC7478355 gene (9,283,876–9,291,377 bp). Therefore, the insertion of T-DNA disrupted the structure of the LOC7478355 gene. According to the National Center for Biotechnology Information (NCBI) analysis, the LOC7478355 gene, which belongs to the *TAFs* gene family, encodes a TAF12 protein, which is one of the core subunits constituting the basic transcription factor TFIID. To understand the impact that this disruption of the gene structure has on the function of this gene, we first analyzed the *TAFs* gene family to clarify the number of genes encoding TAF12 protein in the genome.

We identified 33 *TAFs* genes in the genome of *P. trichocarpa* through bioinformatics analysis. The 33 *PtTAFs* genes were renamed according to their chromosomal positions and the phylogenetic tree constructed with PtTAFs and AtTAFs proteins (Table S3; Fig. S4A). Within the *TAFs* gene family, there are three genes encoding TAF12 protein—*PtTAF12*, *PtTAF12b*, and *PtTAF12c*. Through synteny analysis of the *PtTAFs* gene family, we identified five segmental duplication events involving 10 *PtTAF* genes that encode TAF7, TAF8, and TAF15 proteins. No duplicated segments containing genes encoding TAF12 protein were identified, indicating that *PtTAF12*, *PtTAF12b*, and *PtTAF12c* were not formed from segmental duplication occurring among the three genes (Fig. S4B). The RNA-seq results showed that the expression levels of the three genes in Pb29 leaves were slightly higher than those in poplar 741, but none of the differences were significant, indicating that the transcriptional abundance of the genes encoding TAF12 protein did not change significantly (Fig. 6b).

## Discussion

### Whole-genome resequencing using NGS and Nanopore sequencing improved the accuracy of T-DNA insertion site analysis

Molecular characterization information of T-DNA integration, such as the locations of T-DNA insertion sites and copy numbers, is of great significance for the safety supervision of genetically modified organisms (GMOs) [12]. PCR-based methods are often used to elucidate T-DNA insertion sites and copy numbers. However, these methods are time-consuming, labor-intensive, and produce inaccurate results. When T-DNA integration patterns or the genomes of T-DNA mutants are relatively complex, PCR-based methods cannot be used to accurately determine all T-DNA insertion sites and copy numbers. For example, Gang et al. performed 120 rounds of PCR using 12 border primers and 10 arbitrarily degenerated primers, and located only two T-DNA insertion sites in a birch T-DNA mutant; in contrast, six T-DNA insertion sites were located via genome resequencing using NGS [24]. Whole-genome resequencing is a more effective method for analyzing T-DNA insertion sites and copy numbers. With the emergence and development of high-throughput NGS technology, NGS is now widely used to elucidate T-DNA insertion sites and copy numbers because of its high throughput capability and low cost. However, NGS reads are too short to obtain complete information on the T-DNA insertion sites [25]. In this study, although both NGS and Nanopore sequencing located two T-DNA insertion sites in the Pb29 genome, NGS only detected junction reads on one side of each insertion site. In contrast, complete T-DNA sequences and flanking sequences of T-DNA insertion sites were elucidated using Nanopore sequencing, because it can produce longer reads. Nanopore sequencing can also be used to analyze the entire genome of a T-DNA mutant and identify any chromosomal rearrangements due to T-DNA integration [26]. Therefore, NGS and Nanopore sequencing should be used together to analyze T-DNA mutants, to improve the accuracy of T-DNA insertion site analysis.

### T-DNA insertion sites and copy numbers constitute important molecular information for safety supervision of transgenic plants

**Fig. 7:**
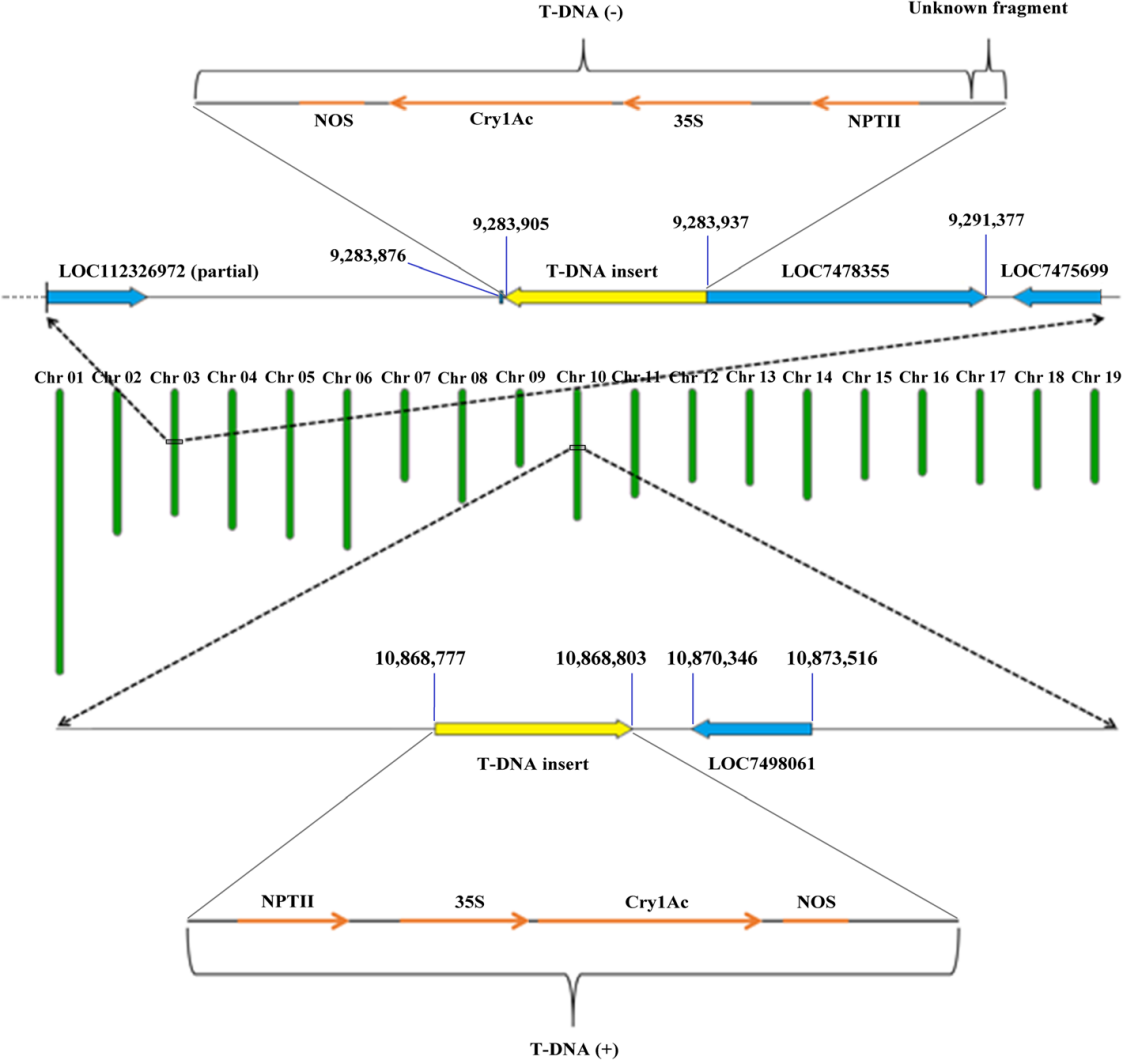
Schematic diagram of T-DNA integration into the Pb29 genome. The green bars represent the 19 chromosomes of poplar with chromosome numbers at the top. The numbers indicate the physical positions of T-DNA or endogenous genes of poplar. (+) represents that T-DNA is inserted in the forward direction. (−) represents that T-DNA is inserted in the reverse direction

There have been several controversial incidents regarding the safety of genetically modified products, such as those involving *Bertholletia excelsa* [27] and the monarch butterfly [28]. Accordingly, the potential threats of genetically modified organisms to the environment and human health are of widespread concern. As a result, many countries have formulated legislation and established agencies to conduct safety assessments and management of genetically modified organisms. The T-DNA insertion site provides important molecular information for the screening and identification processes that are conducted during safety assessments of genetically modified materials, before the materials are released into the environment [29]. Additionally, due to the random location of T-DNA insertion sites and the existence of position effects [30], the euchromatin or heterochromatin region into which the T-DNA is inserted, and the flanking sequences of the T-DNA insertion sites, affect the expression activity of the foreign gene [31, 32]. This activity may also be correlated with the copy number of the foreign gene. For example, the fatty acid content in transgenic rape is positively correlated with the copy number of the thioesterase gene, which encodes an acyl-ACP carrier protein [33]. Furthermore, Cervera et al. found a significant negative correlation between the expression level of the *GUS* gene and its copy number in transgenic citrus [34]. Therefore, T-DNA insertion sites and copy numbers are closely related to the transcription level of the foreign gene, which is important to consider during safety assessments of transgenic plants [12]. In this study, we located two T-DNA insertion sites in the genome of Pb29, a transgenic poplar 741 line, at 9,283,905–9,283,937 bp on Chr03 and 10,868,777–10,868,803 bp on Chr10. According to the sequence information associated with the junction reads, T-DNA was inserted in opposite directions at those two insertion sites. The T-DNA sequences at both insertion sites did not result from tandem duplication; instead, a single-copy integration pattern was observed at both sites (Fig. 7). The T-DNA insertion sites and directions elucidated via resequencing were further confirmed using PCR amplification. At present, the commercialization certificate of transgenic poplar 741 has expired, coupled with China’s very cautious attitude towards the commercialization of genetically modified (GM) plants, which makes the planted area of transgenic insect-resistant poplar in China only 450 ha as of 2011 even if the losses due to insect herbivory to forestry production are continuing to increase [35]. Therefore, the molecular characterization information of T-DNA integration in Pb29 could aid safety supervision and management, and contribute to the reapplication of commercialization certificate and the further extensive commercial planting of Pb29.

### T-DNA and flanking sequence analysis

T-DNA integration into a genome often results in base deletions on the T-DNA left and right border sequences, or to duplications, deletions, and inversions of DNA sequences in the receptor genome [36]; it can even induce chromosomal rearrangement [37]. Kim et al. analyzed a large number of transgenic rice plants and found a difference in the number of base deletions on the T-DNA left and right border sequences, with more deletions occurring on the left side [38]. In a birch T-DNA mutant, the integration of T-DNA led to the deletion or translocation of several chromosomal fragments [21]. Although we did not observe chromosomal rearrangement in the Pb29 genome, base deletions were observed in the T-DNA left and right border sequences, and in the genome sequence at the T-DNA insertion sites; this phenomenon is common in many genetically modified materials. However, we also found a 24-bp fragment that was inserted between the T-DNA insertion site and its right flanking sequence on Chr03. Kersten et al. analyzed two early-flowering poplar lines, T193–2 and T195–1, and found a partial T-DNA fragment with *AtFT*-specific primers at the T-DNA insertion site of each line [8]. However, the difference is that the 24-bp fragment in Pb29 is not part of the T-DNA, so further analysis is needed to elucidate the specific source of the fragment. No *API* gene was detected within the T-DNA sequences at either insertion site, indicating that the *API* gene was not integrated into the Pb29 genome. The two T-DNA sequences are exactly the same, indicating that the *API* gene was not present in the expression vector in *Agrobacterium* before transformation. In addition, the *Agrobacterium tumefaciens* strain LBA4404 used in genetic transformation is deficient in RecA activity (RecA-), indicating that the loss of *API* gene is not caused by homologous recombination, so it is speculated that the *API* gene may be lost during the transformation of the recombinant plasmid into *Agrobacterium*. Therefore, Pb29 is a transgenic line that contains only one insect resistance gene.

### Analysis of the expression levels of genes near the T-DNA insertion sites

T-DNA is randomly inserted into the genome [39, 40]. The introduction of exogenous genes may affect the regulation and expression of endogenous genes in plants [41]. When T-DNA is inserted into a coding gene, the function of the gene is affected. Furthermore, insertion into an intergenic region may affect the expression activity of upstream and downstream genes, resulting in unexpected effects [42]. In the transgenic rice 04Z11EM13 line, T-DNA was inserted into the fourth exon of the *OsBC1L4* gene, resulting in a mutant phenotype that exhibited fewer tillers and dwarfism [43]. Liu et al. analyzed the flanking sequences of the T-DNA insertion site in a rice flag leaf mutant and found that T-DNA insertion led to a significant reduction in the expression of the neighboring AK100376 gene, thus causing phenotypic change [44]. In the genome of the transgenic poplar 741 line Pb29, one T-DNA copy was inserted into the first exon of a gene encoding TAF12 protein, which belongs to the *TAFs* gene family, on Chr03. Through gene family analysis, we identified three genes encoding TAF12 protein in the genome of *P. trichocarpa*. However, the expression of these three genes in Pb29 leaves did not change significantly, which may be due to T-DNA being integrated into only one homologous chromosome, whereas poplar 741 is triploid and has three alleles for each gene. Of the neighboring genes at the two T-DNA insertion sites, except for the LOC7498061 gene, which is located closest to an insertion site, the expression levels of the other four genes did not change significantly in Pb29 leaves, implying that the insertion of T-DNA had little effect on the expression of endogenous genes in the Pb29 genome. Any changes in growth or physiology that may result from the significantly upregulated expression of the LOC7498061 gene in Pb29 need to be studied further.

## Conclusions

In this study, we resequenced the whole genomes of poplar 741 and the transgenic poplar 741 line Pb29 using NGS and Nanopore sequencing. In the Pb29 genome, we found that the T-DNA sequence was inserted inversely into the 9,283,905–9,283,937-bp region on Chr03, and in the forward direction into the 10,868,777–10,868,803-bp region on Chr10. Both insertion sites exhibited a single-copy integration pattern, and the locations and directions of T-DNA insertion were confirmed using PCR amplification. After the T-DNA copies had been inserted into the genome, different degrees of base deletions were detected on the T-DNA left and right border sequences, and in the flanking sequences of the insertion sites. A fragment was found to be inserted between the insertion site and right flanking sequence on Chr03, and no chromosomal rearrangement was detected in the Pb29 genome. Only the *BtCry1Ac* gene was detected in the T-DNA sequence at both insertion sites; no *API* gene was detected, indicating that Pb29 is a transgenic line containing only one insect resistance gene. The insertion of T-DNA destroyed the structure of a gene encoding TAF12 protein on Chr03, but the transcriptional abundance of this gene did not change significantly in Pb29 leaves. Except for the LOC7498061 gene, which is located closest to a T-DNA insertion site, the expression of four other neighboring genes did not change significantly in Pb29 leaves. This study provides molecular characterization information of T-DNA integration in transgenic poplar 741 line Pb29, which contribute to safety supervision and further extensive commercial planting of transgenic poplar 741.

## Methods

### Plant materials

The experimental materials used in this study were tissue culture seedlings of poplar 741 and transgenic poplar 741 line Pb29, which were stored in Hebei Key Laboratory of Forest Germplasm Resources and Forest Protection, College of Forestry, Hebei Agricultural University. The leaves of poplar 741 and Pb29 were collected, immediately placed into liquid nitrogen, and preserved at − 80° for subsequent DNA extraction.

### Genome resequencing using NGS and data analysis

Genome resequencing of Pb29 via NGS was performed by Biomics Co., Ltd. (Beijing, China). Genomic DNA was extracted using a plant DNA extraction kit (SENO Biological Technology Co., Ltd., Zhangjiakou, China) in accordance with the manufacturer’s protocol and quantified using a NanoDrop 2000 spectrophotometer (Thermo Fisher Scientific, Waltham, MA, USA). The DNA was broken into fragments with an average length of 300 bp to construct the library. The library was then sequenced using the HiSeq sequencing platform (Illumina, San Diego, CA, USA), and 150-bp paired-end reads were generated. After sequencing, the raw data were initially screened to remove adapter sequences and low-quality reads (Q < 20) and thus obtain clean, high-quality data. Using AIM-HII software [45], the clean reads were compared against the *Populus trichocarpa* genome sequence and the vector sequence to identify junction reads. First, the junction reads that aligned with both the reference genome sequence and the vector sequence were identified, and the T-DNA insertion sites and directions were then determined based on the alignment information associated with the junction reads.

### PCR verification of T-DNA insertion sites and directions

To verify the T-DNA insertion sites and directions, we designed primers based on the flanking sequences of the T-DNA insertion sites and the T-DNA sequence (Table S4), and amplified the genomic DNA of poplar 741 and Pb29. The PCR products corresponding to the sides of the junction reads undetected by NGS were purified and ligated into the pUCm-T vector. After 12 h at 16 °C, the plasmid was transformed into *E. coli* DH5α competent cells. The cells were shaken for 1 h in a constant-temperature shaker at 37 °C, and then plated onto a Luria-Bertani agar plate containing ampicillin and cultured for 8 h at 37 °C. Single colonies were selected and sent to Beijing Zhongke Xilin Biotechnology Co., Ltd. (Beijing, China) for sequencing, to determine the integrity of the flanking sequences.

### Genome resequencing using Nanopore sequencing and data analysis

The genomic DNA of poplar 741 and Pb29 was resequenced using the Nanopore sequencing platform (Biomarker Technologies, Beijing, China). After extracting the genomic DNA of poplar 741 and Pb29, the purity, concentration, and integrity of the extracted DNA were inspected using a NanoDrop spectrophotometer, Qubit fluorometer (Invitrogen, Carlsbad, CA, USA), and 0.35% agarose gel electrophoresis, respectively. After passing the quality checks, the DNA samples were used to construct libraries and sequenced with Ligation Sequencing Kit 1D (SQK-LSK109; Oxford Nanopore Technologies, Oxford, UK). Low-quality reads, reads with adapters, and short sequencing reads (length < 500 bp) were filtered from the raw reads. Then, Minimap2 software (https://github.com/lh3/minimap2) [46] was used to compare the clean reads with the reference genome and vector sequences (at the same time). The junction reads thus obtained were saved in BAM file format, and the data were visualized with IGV software (http://www.broadinstitute.org/software/igv/) to locate the T-DNA insertion sites. By comparing the clean reads and reference genome sequence, information such as alignment rate and sequencing depth and coverage could be calculated. Sniffles software [47] was used to detect large SVs in the genome, such as insertions, deletions, repetitions, inversions, and translocations, and the SV distribution was visualized using Circos software (http://circos.ca) [48].

### T-DNA and flanking sequence analysis

Part of the flanking sequences were isolated from the junction reads obtained by NGS and the Sanger sequencing reads obtained from the PCR products; the other part of the flanking sequences and complete T-DNA sequences were extracted from the junction reads obtained by genome resequencing using Nanopore sequencing. All flanking and T-DNA sequences were compared with the genome and vector sequences, respectively, to determine the integrity of the flanking and T-DNA sequences at the insertion sites.

### RNA-sequencing (RNA-Seq) analysis of the expression levels of genes located near the insertion sites

To detect whether the insertion of T-DNA affects the expression of upstream and downstream genes near the insertion site, we analyzed poplar 741 and Pb29 leaves using RNA-seq. First, healthy and mature leaves along a long branch within the upper parts of mature trees of poplar 741 and Pb29 that have been grown for 6 years in the test forest were collected. Then, total RNA was extracted from the leaves using a plant RNA extraction kit (SENO Biological Technology Co., Ltd.) in accordance with the manufacturer’s instructions. The concentration and quality of the RNA samples were determined using a NanoDrop 2000 spectrophotometer and an Agilent 2100 Bioanalyzer (Agilent Technologies, Santa Clara, CA, USA). After the quality of the RNA samples had been verified, a cDNA library of each sample was constructed and Illumina sequencing was performed by LC Bio Technology Co., Ltd. (Hangzhou, China). FPKM values were used to examine changes in the expression of genes upstream and downstream of the T-DNA insertion sites. Three biological replicates for each poplar line were sampled.

### Identification of *TAFs* gene family members and expression of genes encoding TAF12 protein

The whole-genome and protein sequences of *P. trichocarpa* were downloaded from the NCBI database (https://www.ncbi.nlm.nih.gov/genome/98). Identified TAFs protein sequences from *A. thaliana* (downloaded from the Arabidopsis Information Resource; https://www.arabidopsis.org/) were used as queries in BLASTP searches against the *P. trichocarpa* genome with an e-value cutoff of 1e-10. Redundant sequences were manually removed, and all candidate proteins were analyzed and verified using InterProScan (http://www.ebi.ac.uk/interpro/search/sequence-search) and the Conserved Domains Database (https://www.ncbi.nlm.nih.gov/cdd). A multiple sequence alignment of TAFs proteins was generated using ClustalW in MEGA 7 (https://www.megasoftware.net) with default parameters. A neighbor-joining phylogenetic tree was constructed based on the alignment results with the following settings: Poisson model, pairwise deletion, and 1000 bootstrap replications. *PtTAFs* gene duplication events were analyzed using the Multiple Collinearity Scan toolkit (MCScanX; http://chibba.pgml.uga.edu/mcscan2) [49]. The expression levels of the genes encoding TAF12 protein in leaves were analyzed using the above-mentioned RNA-seq.

## Supplementary Information

**Additional file 1: Table S1.** The summary of sequence data from NGS.

**Additional file 2: Table S2.** The junction reads obtained by NGS in the Pb29 genome.

**Additional file 3: Table S3.** The physical characteristics of *TAFs* gene family in *Populus trichocarpa*. 

**Additional file 4: Table S4.** Primer sequences for verifying T-DNA insertion sites.

**Additional file 5: Figure S1. **Genomewide distribution of read coverage of poplar 741 and Pb29.

**Additional file 6: Figure S2. **The distribution of SV variants on chromosomes in genomes of poplar 741 and Pb29. From outside to inside: chromosome coordinates (Mb), insertion, deletion, inversion, duplication and translocation.

**Additional file 7: Figure S3. **Partial alignment result of sequence data of poplar 741 with *P. trichocarpa* genome.

**Additional file 8: Figure S4. **Phylogentic analysis (a) and synteny analysis (b) of PtTAFs family genes.

## Data Availability

The datasets analysed during the current study are available in the Sequence Read Archive (SRA, https://www.ncbi.nlm.nih.gov/sra) under accession number PRJNA720519 and PRJNA720721.

## Abbreviations

PCR: Polymerase chain reaction
NGS: Next-generation sequencing
IGV: Integrative Genomics Viewer
SV: Structural variation
FPKM: Fragments Per Kilobase per Million
NCBI: National Center for Biotechnology Information
GMOs: Genetically modified organisms
GM: Genetically modified
MCScanX: Multiple Collinearity Scan toolkit
