# Supplementary material for: Whole-genome resequencing using next-generation and Nanopore sequencing for molecular characterization of T-DNA integration in transgenic poplar 741
Source: BMC Genomics. 2021 May 6;22:329. doi: 10.1186/s12864-021-07625-y (PMC8101135; doi:10.1186/s12864-021-07625-y)
Supplement: Supplementary file 1 — Additional file 1: Table S1. The summary of sequence data from NGS. [file 12864_2021_7625_MOESM1_ESM.doc]

| Clean reads | Clean bases (Gb) | GC (%) | Q20(%) | Q30(%) |
| --- | --- | --- | --- | --- |
| 52,313,447 | 15.7 | 37.42 | 97.36 | 92.77 |

**Table S1** The summary of sequence data from NGS.
