# Supplementary material for: Whole-genome resequencing using next-generation and Nanopore sequencing for molecular characterization of T-DNA integration in transgenic poplar 741
Source: BMC Genomics. 2021 May 6;22:329. doi: 10.1186/s12864-021-07625-y (PMC8101135; doi:10.1186/s12864-021-07625-y)
Supplement: Supplementary file 2 — Additional file 2: Table S2. The junction reads obtained by NGS in the Pb29 genome. [file 12864_2021_7625_MOESM2_ESM.doc]

Table S2. The junction reads obtained by NGS in the Pb29 genome.

| Kinds | Sequences (5'-3', Black: Genome sequence; Red: Vector sequence; Highlight: Unknown fragment sequence |
| --- | --- |
| Chr03-read1 | TCTGACGTATGTGCTTAGCTCATTAAACTCCAGAAACCCGCGGCTGAGTGGCTCCTTCAACGTTGCGGTTCTGTCAGTTCCAAACGTAAAACGGCTTGTCCCGCGTCATCGGCGGGGGTCATAACGTGACTCCCTTAATTCTCCGCTCATGATCAGATTGTCGTTTCCCGCCTTCAGTTTAAACTATCAGTGTCTAAGCGTCAATTTGTTTACACCAAATTCATCGAAAGAATGAATTTTTAAGTAAAATTAAGAAAA |
| Chr03-read2 | GACGTATGTGCTTAGCTCATTAAACTCCAGAAACCCGCGGCTGAGTGGCTCCTTCAACGTTGCGGTTCTGTCAGTTCCAAACGTAAAACGGCTTGTCCCGCGTCATCGGCGGGGGTCATAACGTGACTCCCTTAATTCTCCGCTCATGATCAGATTGTCGTTTCCCGCCTTCAGTTTAAACTATCAGTGTCTAAGCGTCAATTTGTTTACACCAAATTCATCGAAAGAATGAATTTTTAAGTAAAATTAAGAA |
| Chr03-read3 | CTGTCAGTTCCAAACGTAAAACGGCTTGTCCCGCGTCATCGGCGGGGGTCATAACGTGACTCCCTTAATTCTCCGCTCATGATCAGATTGTCGTTTCCCGCCTTCAGTTTAAACTATCAGTGTCTAAGCGTCAATTTGTTTACACCAAATTCATCGAAAGAATGAATTTTTAAGTAAAATTAAGAAAACCCATAAATCTTC |
| Chr03-read4 | TCTCCGCTCATGATCAGATTGTCGTTTCCCGCCTTCAGTTTAAACTATCAGTGTCTAAGCGTCAATTTGTTTACACCAAATTCATCGAAAGAATGAATTTTTAAGTAAAATTAAGAAAACCCATAAATCTTCGGTTCCAACCCAATCCAA |
| Chr03-read5 | CTCATGATCAGATTGTCGTTTCCCGCCTTCAGTTTAAACTATCAGTGTCTAAGCGTCAATTTGTTTACACCAAATTCATCGAAAGAATGAATTTTTAAGTAAAATTAAGAAAACCCATAAATCTTCGGTTCCAACCCAATCCAATCCATG |
| Chr03-read6 | CTCATGATAAGATTGTCGTTTCCCGCCTTAAGTTTAAACTATCAGTGTCTAAGCGTCTATTTGTTTACACCAAATTCATCGAAAGATTGAATTTTTTAGTAAATTTAAGAAAACCCATAACTCTTCGGTTCCAACCCATTCCAATCCAGG |
| Chr03-read7 | TCCCGCGTCATCGGCGGGGGTCATAACGTGACTCCCTTAATTCTCCGCTCATGATCAGATTGTCGTTTCCCGCCTTCAGTTTAAACTATCAGTGTCTAAGCGTCAATTTGTTTACACCAAATTCATCGAAAGAATGAATTTTTAAGTAAAATTAAGAAAACCCATAAATCTTCGGTTCCAACCCAATCCAATCCATGGCAGAAAATGCAGCTGCTGCT |
| Chr03-read8 | CCGCTCATGATCAGATTGTCGTTTCCCGCCTTCAGTTTAAACTATCAGTGTCTAAGCGTCAATTTGTTTACACCAAATTCATCGAAAGAATGAATTTTTAAGTAAAATTAAGAAAACCCATAAATCTTCGGTTCCAACCCAATCCAATCCATGGCAGAAAATGCAGCTGCTGCTGCTGCAGCCTCTTCGCCTAAACCTCTCCCTTCT |
| Chr03-read9 | TCAGTGTCTAAGCACAATTTGTTTACACCATCAGATTGTCGTTTCCCGCCTTCAGTTTAAACTATCAGTGTCTAAGCGTCAATTTGTTTACACCAAATTCATCGAAAGAATGAATTTTTAAGTAAAATTAAGAAAACCCATAAATCTTCGGTTCCAACCCAATCCAATCCATGGCAGAAAATGCAGCTGCTGCTGCTGCAGCCTCTTCGCCTAAACCTCTCCCTTCTCCGAATACAATTATAGACCCATCGTCACAACCACAACC |
| Chr10-read1 | CCTATCTGTGTAGGCTGAAAAAAATAGTAGCCTATAATGTGGGCCGTACGCCCGCTGGCAGGGAGGCGCCGTACTAAATGAATCGTGTCATTATCTGGAGGGCCGGGGACTGATTATTACTTACCTGTACTGGAAATATTTATAAGATGGGGACATTTTAGCTGCTAGAATATAGTTTAAACTGAAGGCGGGAAACGACAATCTGATCATGAG |
| Chr10-read2 | ATTATCTGGAGGGCCGGGGACTGATTATTACTTACCTGTACTGGAAATATTTATAAGATGGGGACATTTTAGCTGCTAGAATATAGTTTAAACTGAAGGCGGGAAACGACAATCTGATCATGAGCGGAGAATTAAGGGAGTCACGTTATG |
| Chr10-read3 | GAAATATTTATAAGATGGGGACATTTTAGCTGCTAGAATATAGTTTAAACTGAAGGCGGGAAACGACAATCTGATCATGAGCGGAGAATTAAGGGAGTCACGTTATGACCCCCGCCGATGACGCGGGACAAGCCGTTTTACGTTTGGAAC |
| Chr10-read4 | AAATATTTATAAGATGGGGACATTTTAGCTGCTAGAATATAGTTTAAACTGAAGGCGGGAAACGACAATCTGATCATGAGCGGAGAATTAAGGGAGTCACGTTATGACCCCCGCCGATGACGCGGGACAAGCCGTTTTACGTTTGGAACT |
