# Supplementary material for: Whole-genome resequencing using next-generation and Nanopore sequencing for molecular characterization of T-DNA integration in transgenic poplar 741
Source: BMC Genomics. 2021 May 6;22:329. doi: 10.1186/s12864-021-07625-y (PMC8101135; doi:10.1186/s12864-021-07625-y)
Supplement: Supplementary file 3 — Additional file 3: Table S3. The physical characteristics of TAFs gene family in Populus trichocarpa. [file 12864_2021_7625_MOESM3_ESM.doc]

**Table S3** The physical characteristics of *TAFs* gene family in *Populus trichocarpa*.

| Name | Gene symbol | Chr | Genomic location | Name | Gene symbol | Chr | Genomic location |
| --- | --- | --- | --- | --- | --- | --- | --- |
|
| *TAF1* | LOC7486075 | 7 | 13532968:13553923 | *TAF9* | LOC7478099 | 1 | 14186976:14189156 |
| *TAF1b* | LOC7472543 | 17 | 3964494:3986991 | *TAF10* | LOC7465449 | 18 | 1025682:1027995 |
| *TAF2* | LOC18105528 | 15 | 5635684:5667456 | *TAF11* | LOC7491456 | 1 | 5828629:5834085 |
| *TAF4* | LOC7462851 | 2 | 8999445:9006794 | *TAF11b* | LOC112328249 | 7 | 19133:20885 |
| *TAF4b* | LOC7497338 | 14 | 1709740:1716465 | *TAF12* | LOC7478108 | 1 | 14349460:14356956 |
| *TAF5* | LOC7489149 | 6 | 26638264:26645300 | *TAF12b* | LOC7478355 | 3 | 9283876:9291377 |
| *TAF5b* | LOC7465413 | 18 | 1568484:1574762 | *TAF12c* | LOC7454916 | 6 | 6315941:6324206 |
| *TAF6* | LOC7487521 | 1 | 36978924:36984663 | *TAF13* | LOC7495223 | 14 | 9488112:9491114 |
| *TAF6b* | LOC7468541 | 14 | 12685250:12694053 | *TAF14* | LOC7453610 | 4 | 18542675:18546560 |
| *TAF7* | LOC7457707 | 1 | 423095:426480 | *TAF14b* | LOC7480178 | 7 | 991034:993419 |
| *TAF7b* | LOC7463581 | 3 | 21459132:21462376 | *TAF14c* | LOC7494288 | 9 | 10457158:10460675 |
| *TAF8* | LOC7480583 | 4 | 10747129:10749249 | *TAF15* | LOC18095136 | 1 | 28789532:28794606 |
| *TAF8b* | LOC18100163 | 6 | 10071150:10074496 | *TAF15b* | LOC18100420 | 6 | 15177976:15183124 |
| *TAF8c* | LOC18103895 | 12 | 13966349:13966864 | *TAF15c* | LOC7498166 | 8 | 5359361:5362704 |
| *TAF8d* | LOC7457637 | 15 | 13330434:13331563 | *TAF15d* | LOC18102067 | 9 | 7426288:7431562 |
| *TAF8e* | LOC7486490 | 16 | 7582005:7583848 | *TAF15e* | LOC7481594 | 10 | 17229566:17233844 |
| *TAF8f* | LOC7484577 | 17 | 11185460:11187491 |  |  |  |  |
