# Supplementary material for: Whole-genome resequencing using next-generation and Nanopore sequencing for molecular characterization of T-DNA integration in transgenic poplar 741
Source: BMC Genomics. 2021 May 6;22:329. doi: 10.1186/s12864-021-07625-y (PMC8101135; doi:10.1186/s12864-021-07625-y)
Supplement: Supplementary file 4 — Additional file 4: Table S4. Primer sequences for verifying T-DNA insertion sites. [file 12864_2021_7625_MOESM4_ESM.doc]

**Table S4** Primer sequences for verifying T-DNA insertion sites.

| Primer name | Sequences (5' to 3') |
| --- | --- |
| 131#S5F | TAGTGACCTTAGGCGACTTTTGAACG |
| 131#S2F | ATTTGGGTGATGGTTCACGTAGTGG |
| Chr3u-F1 | AGAGTACGCCCTTTGATTATTTGCT |
| Chr3d-R2 | GCCTGACATTGCGGTGACATTCTGC |
| Chr10u-F2 | CGACGAGATGCCTCCACCATTCTGA |
| Chr10d-R2 | TCTTCTATGGTTGCTCCTGCTTTGT |
