# Supplementary figures and images for: Whole-genome resequencing using next-generation and Nanopore sequencing for molecular characterization of T-DNA integration in transgenic poplar 741
Source: BMC Genomics. 2021 May 6;22:329. doi: 10.1186/s12864-021-07625-y (PMC8101135; doi:10.1186/s12864-021-07625-y)

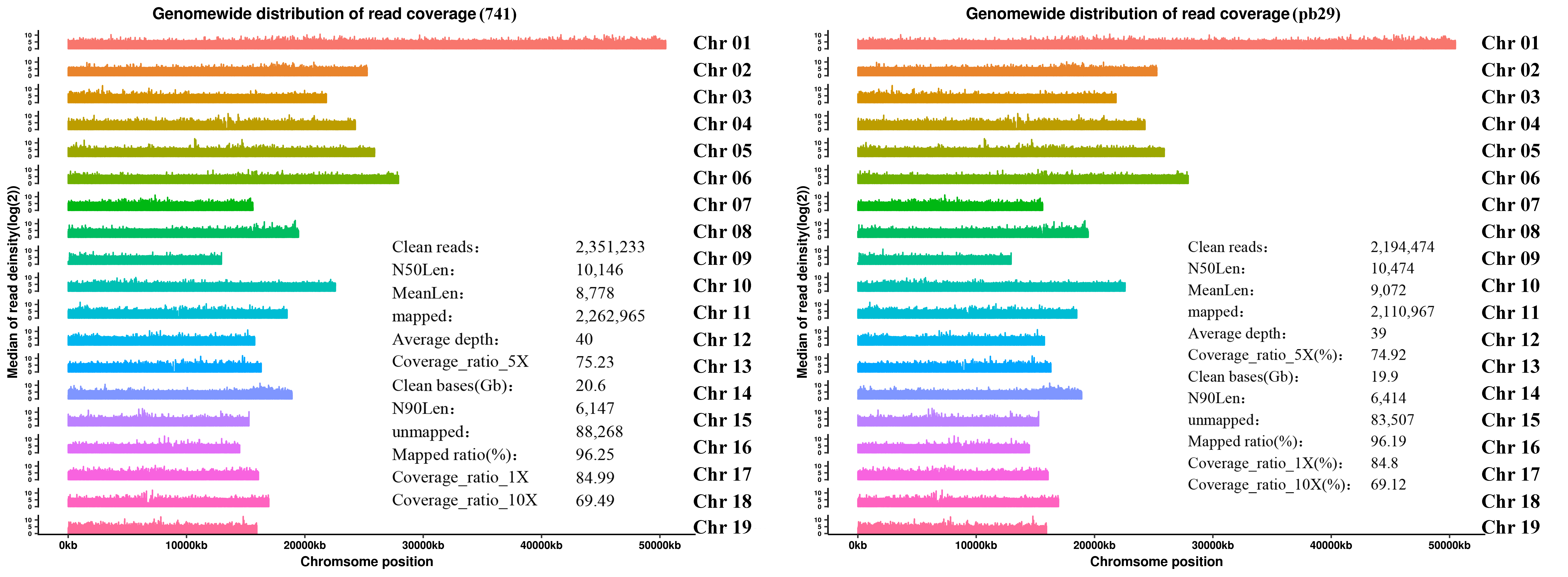

Supplement: Supplementary file 5 — Additional file 5: Figure S1. Genomewide distribution of read coverage of poplar 741 and Pb29. [file 12864_2021_7625_MOESM5_ESM.png]

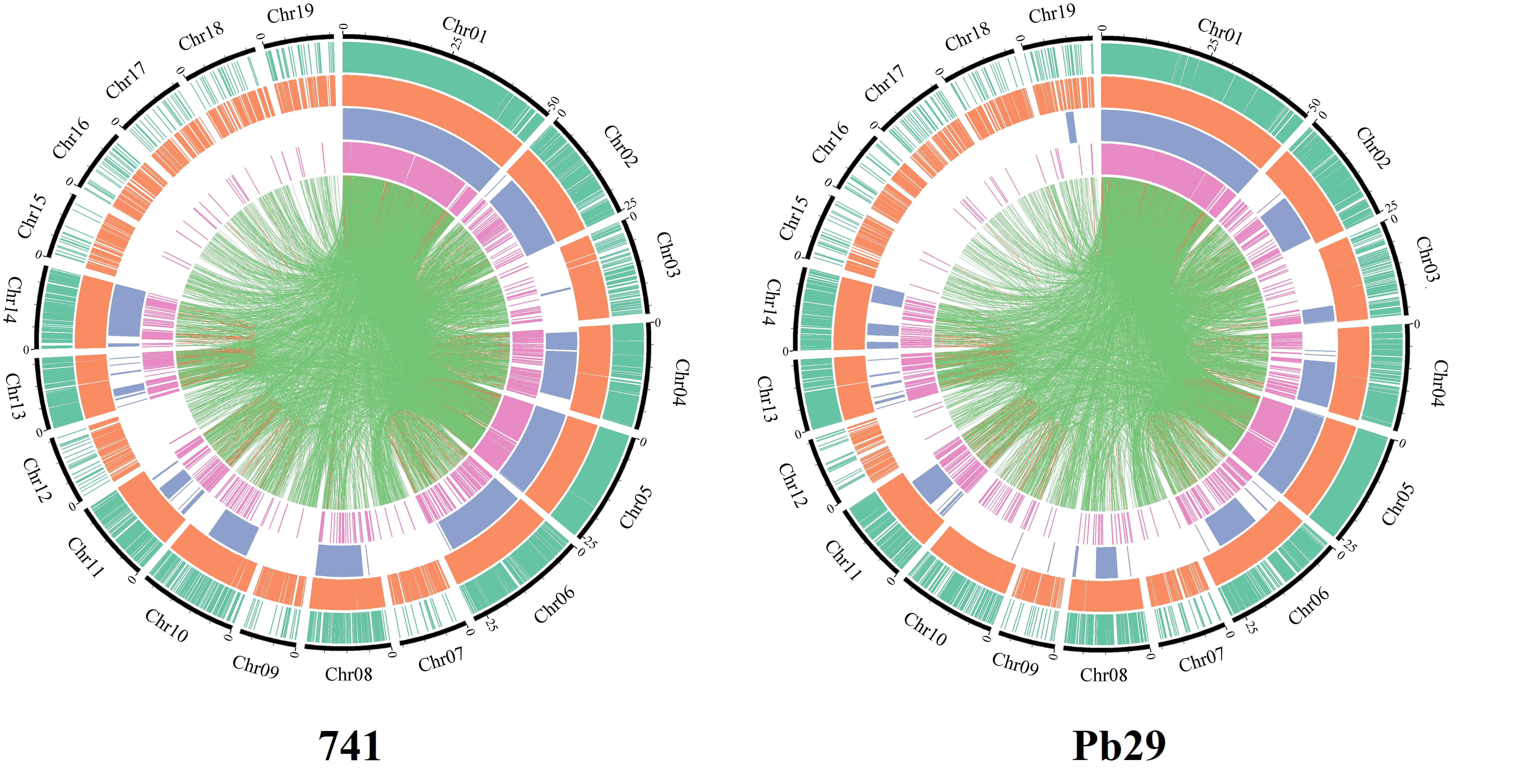

Supplement: Supplementary file 6 — Additional file 6: Figure S2. The distribution of SV variants on chromosomes in genomes of poplar 741 and Pb29. From outside to inside: chromosome coordinates (Mb), insertion, deletion, inversion, duplication and translocation. [file 12864_2021_7625_MOESM6_ESM.png]

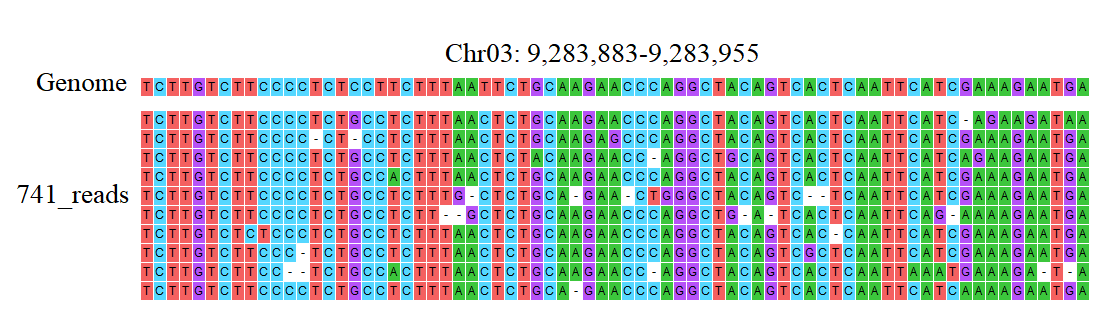

Supplement: Supplementary file 7 — Additional file 7: Figure S3. Partial alignment result of sequence data of poplar 741 with P. trichocarpa genome. [file 12864_2021_7625_MOESM7_ESM.png]

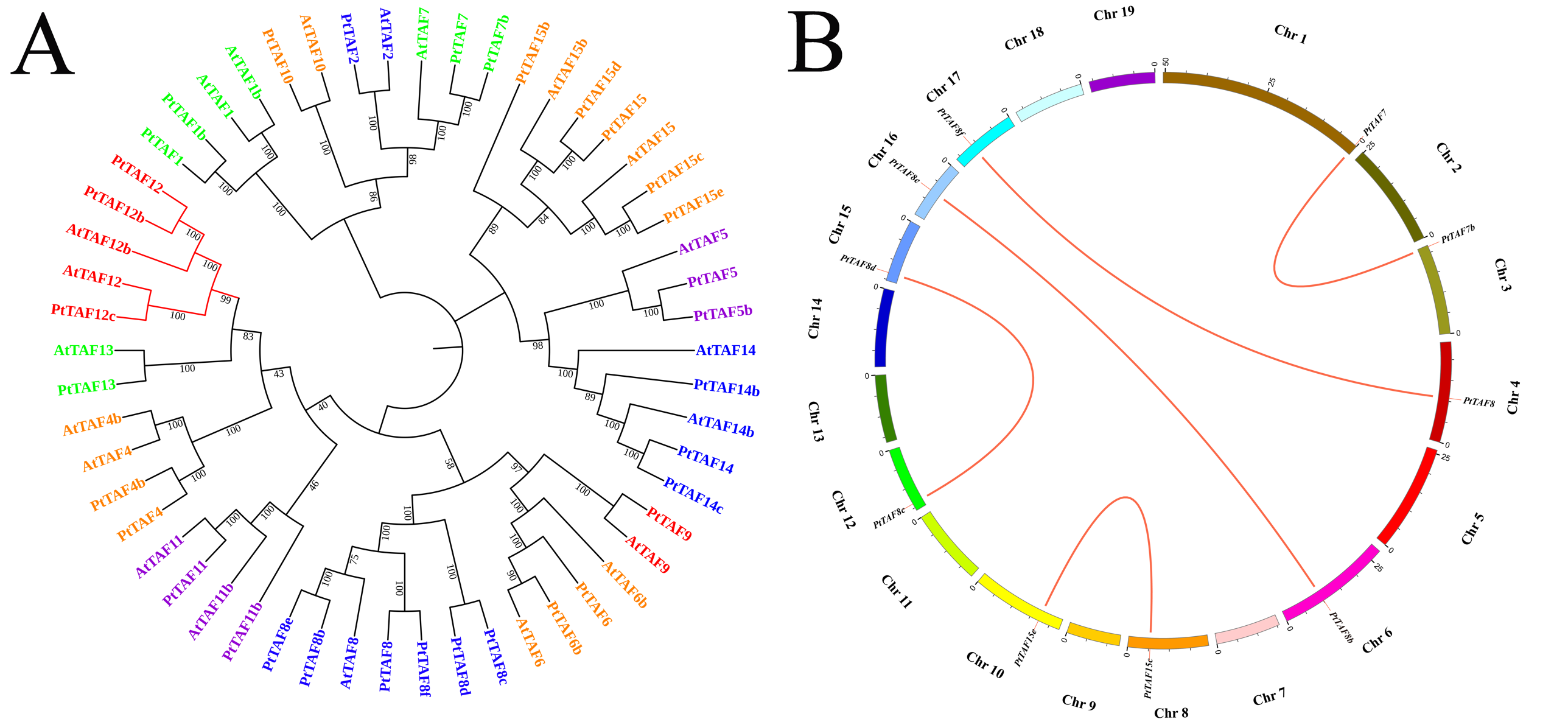

Supplement: Supplementary file 8 — Additional file 8: Figure S4. Phylogentic analysis (a) and synteny analysis (b) of PtTAFs family genes. [file 12864_2021_7625_MOESM8_ESM.png]
